# *Toxoplasma gondii* Modulates the Host Cell Responses: An Overview of Apoptosis Pathways

**DOI:** 10.1155/2019/6152489

**Published:** 2019-04-04

**Authors:** Nour Mammari, Mohamad Adnan Halabi, Souha Yaacoub, Hilda Chlala, Marie-Laure Dardé, Bertrand Courtioux

**Affiliations:** ^1^UMR_S 1094, Tropical Neuroepidemiology, Institute of Neuroepidemiology and Tropical Neurology, Université de Limoges, CNRS FR 3503 GEIST, 87000 Limoges, France; ^2^Medical Laboratory Department, Holy Family University, 5534 Batroun, Lebanon; ^3^Mixed Research Unit National Center for Scientific Research 7276, INSERM U1262, Control of Immune Response B and Lymphoproliferation, University of Limoges, 87000 Limoges, France; ^4^Department of Parasitology and Biological Resource Centre for Toxoplasma, University Hospital, 87000 Limoges, France

## Abstract

Infection with* Toxoplasma gondii *has a major implication in public health.* Toxoplasma gondii *is an obligate intracellular protozoan parasite that can infect all nucleated cells belonging to a wide range of host species. One of the particularities of this parasite is its invasion and persistence in host cells of immunocompetent people. This infection is usually asymptomatic. In immunocompromised patients, the infection is severe and symptomatic. The mechanisms by which* T. gondii* persists are poorly studied in humans. In mouse models, many aspects of the interaction between the parasite and the host cells are being studied. Apoptosis is one of these mechanisms that could be modulated by* Toxoplasma* to persist in host cells. Indeed,* Toxoplasma* has often been implicated in the regulation of apoptosis and viability mechanisms in both human and murine infection models. Several of these studies centered on the regulation of apoptosis pathways have revealed interference of this parasite with host cell immunity, cell signalling, and invasion mechanisms. This review provides an overview of recent studies concerning the effect of* Toxoplasma *on different apoptotic pathways in infected host cells.

## 1. Introduction

**Figure 1:**
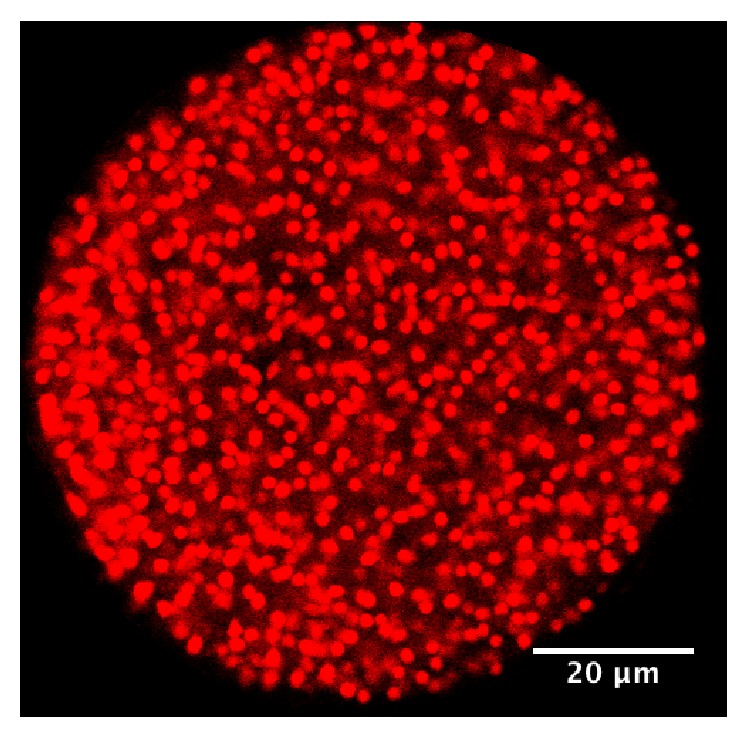
Cyst of* Toxoplasma gondii* containing bradyzoites.

Toxoplasmosis is known to be one of the most common infections worldwide in humans and warm-blooded animals. One-third of the world's population is infected with toxoplasmosis [1]. This parasite is able to theoretically infect all warm-blooded animals from birds to mammals. Cats, or more generally* Felidae*, are the only animals that allow for the sexual reproduction leading to oocyst excretion in their feces. Infection occurs by ingestion of cysts contained in parasitized meat or oocysts present in vegetables contaminated by cat feces. After an initial stage of tachyzoite proliferation and dissemination, the infection usually persists in a chronic form, following the formation of tissue cysts containing bradyzoites, especially in the brain [2] (Figure 1).

Clinical forms of toxoplasmosis in humans range from asymptomatic to lethal depending on the host's immune response. Since its entry into the host,* Toxoplasma gondii (T. gondii)* triggers an immune response orchestrated by interferon-gamma (IFN-*γ*) secreted by T lymphocytes and natural killer cells (NK).

In the case of immunocompetent hosts, the immune response usually leads to the acquisition of protective immunity, preventing any reinfection. In case of immune deficiency, particularly in patients with acquired immune deficiency syndrome (AIDS), the bradyzoites released following cyst rupture are converted into tachyzoites, the proliferation of which is not effectively controlled by the immune response of the host, leading to encephalitis [3].

Most protozoan parasites can modulate the host cell response and apoptosis is one of the mechanisms targeted by these parasites. Apoptosis or programmed cell death is the process by which cells trigger their destruction. It was firstly described by Kerr et al. [4] and is an essential pathway for development and tissue homeostasis.

Unlike necrosis, no inflammatory reaction is involved during apoptosis, and the membrane integrity is preserved. This phenomenon is tightly regulated, and any imbalance can cause diseases. While excessive apoptosis can lead to degenerative diseases, a defect in apoptosis can lead to the development of autoimmune diseases or participate in carcinogenesis.

Apoptosis can be induced by many stimuli (growth factor deprivation, exposure to ultraviolet rays, or exogenous factors such as cancer). According to these signals, we can distinguish two major signalling pathways: the death receptor and the mitochondrial pathways. In addition, study of Ran and collaborators describes that* T. gondii* can stimulate apoptosis via endoplasmic reticulum (ER) stress during toxoplasmic encephalitis (TE) [5].

Most proapoptotic stimuli are associated with a permeabilization of the outer mitochondrial membrane. This process is regulated directly or indirectly by BH3- only molecules leading to the release of cytochrome c into the cytosol. Cytochrome c promotes the activation of caspases, the proteases responsible for the execution of apoptosis [6].

*Toxoplasma gondii *has been shown to modulate apoptotic responses of host cells to survive in infected cells. This differs depending on the cell types.* T. gondii *protects different cell types from apoptosis induced by a variety of proapoptotic treatments [7]. Blocking apoptosis helps the parasite to avoid rapid clearance by infected cells, which is activated by signals emitted by apoptotic cells. In contrast,* T. gondii *can initiate apoptosis in some cells and during specific stages of infection.

In PubMed, less than 100 articles have been published since 2014 on this subject. Herein we provide an overview of studies centered on the effect of* T. gondii *on the different apoptosis pathways to invade and persist in host cells.

## 2. *Toxoplasma gondii *Modulates Pathways to Invade Host Cells

Different studies performed in murine and in human cells showed that* T. gondii *modulates pathways during host invasion in a way that could have an impact on host cell apoptosis. In mice, tachyzoite proteins can react either directly by disrupting the host cell's immune process or indirectly by affecting the regulation of the transcriptomic process* in vitro* [8, 9]. In human promyelocytic leukemia cells (HL-60) and human histiocytic lymphoma cells (U937), studies have shown that* Toxoplasma* which resides within the host cell in a parasitophorous vacuole (PV) can inhibit or initiate apoptosis [10]. Parasitophorous vacuole formation results from the sequential secretion of parasite secretory organelles called micronemes (MIC), rhoptries (ROP), and dense granules (GRA) [11]. These parasite-derived effectors are deeply involved in virulence and apoptosis modulation. A prominent feature of the PV is the presence of an intravacuolar network (IVN) that connects the parasites to each other and to the PV membrane [12]. A study by Lopez and collaborators shows that the IVN may have a role in immune modulation in the murine model [13]. However, the GRA6 association with the PV membrane alone promotes major histocompatibility complex I (MHCI) antigen presentation to active CD8 T cells, but the presence of the IVN limits this action. This result is a new way to understand the immunopathogenicity regulation used by* T. gondii *[13].

A notable feature of* T. gondii *is its ability to invade and maintain host cell viability at any time during its intracellular residence [14]. In fact, it is known that proapoptotic molecules such as nitrite oxide (NO) or tumor necrosis factor *α* (TNF*α*) are produced by macrophages in response to* T. gondii *invasion [15, 16]. Exogenous NO can indeed induce egress of* T. gondii *tachyzoites from mouse peritoneal macrophages, [17] and IFN-*γ* can promote tachyzoite egress in murine astrocytes at 3 h after the infection via a mechanism that involves the interferon-regulated GTPase (IRG) protein Irgm3 [18].

In mice, the majority of tachyzoite-secreted molecules are released during invasion [9]. A molecule secreted by the rhoptries of the parasite, ROP16, can perform a tyrosine phosphorylation of the signal transducer and activator of transcription STAT3 and STAT6 protein [19–21]. Alternatively, ROP16 can induce activation of macrophages at an early phase of infection by stimulating IL-4 and IL-10 expression, thus promoting a Th2- response [9, 22].

Additional effectors secreted by other parasitic organelles, the dense granules GRA15 and GRA16, are released and traffic to the host cell [23, 24], positively regulating genes responsible for the cell cycle and p53 tumor suppressor pathway [23]. GRA15 has been shown to regulate also host nuclear factor NF-*κ*B pathways. It affects both parasite growth and cytokine levels specifically in* Toxoplasma* type II infection [22]. GRA15 has been implicated in activation of the proapoptotic pathway, activation of macrophages, and stimulation of Th1-response immunity, subsequently activating NK and Th17 cells resulting in oxidative stress [22, 25]. A recent study has revealed that GRA15 induces trophoblast apoptosis* in vitro* and causes abnormal pregnancies in mouse models [26].

*In vivo* GRA7 has a protective role in* T. gondii* infection. It induces NF-*κ*B signaling activation during immune responses through formation of a complex with MyD88. GRA7/MyD88 complex-dependent NF-*κ*B activation promotes the activation of TNF receptor-associated factor 6 (TRAF6) and reactive oxygen species (ROS) generation. Activation of this pathway enhances the release of inflammatory mediators, resulting in crucial protective efficacy against* T. gondii *infection* in vivo* [27]. The polymorphic rhoptry protein ROP18 is a key serine/threonine kinase that phosphorylates host proteins to modulate acute virulence, by providing a survival advantage to the infectious agent. The kinase activity of ROP18 suppresses NF-*κ*B activation through the promotion of p65 degradation and proinflammatory cytokine suppression, in type I strains* T. gondii* [28]. A recent study shows that ROP18 can phosphorylate ER-associated protein called reticulon 1-C (RTN1-C) and promote ER stress-mediated apoptosis in neural cells in murine model [5].

To resist the parasite, a strong Th1 immune response and interleukin (IL)-12 activation is needed [29, 30]. This immune mechanism is based on stimulation NK and T cells, IFN-*γ* production, and activation of interferon-regulated GTPases (IRGs) to kill the parasite in the murine model [8]. However, in human cells,* Toxoplasma* utilizes iNOS to antagonize IFN-*γ* induced IDO1-mediated immunity against GRA15 effector which benefits parasite growth [25]. ROP16 has been implicated in the regulation of this immune response. ROP16 can stimulate secretion of a STAT3/6-dependent macrophage response that can reduce IL-12p70 synthesis [20]. Recently, another effect of ROP16 has been described, which has to do with regulation of tumor suppressor protein p53 gene transcription and translation in the human neuroblastoma cell line SH-SY5Y [31], in which ROP16 can induce activation of p53 protein, which promotes cell cycle arrest and p53-dependent apoptosis activation [32].

Another signaling pathway responsible for control of IL-12p70 synthesis is the mitogen-activated protein kinase (MAPKinase) pathway. MAPKinase pathway activation involves a series of kinases that autophosphorylate and activate each other (MAP4K, MAP3K, and MAP2K), culminating with the phosphorylation of a specific MAPK that regulates the expression of a subset of genes through activation of specific transcription factors. It has been reported that* Toxoplasma* is dependent on MAPKinase kinase (MKK) to induce autophosphorylation of the p38-*α* MAPK and to produce IL-12p70 [33]. In a Th1 immune response, IL-23 and IL-12 are the most important mediators to bridge innate and adaptive immunity. In* T. gondii *RH strain-infected THP-1 cells (human monocyte cells), IL-23 production is positively regulated by Phosphoinositide 2-Kinase (PI3K) and MAPK but negatively regulated by P38 MAPK. In contrast, IL-12 production is negatively regulated by PI3K and positively regulated by P38 MAPK and c-Jun N-terminal kinase (JNK) [34].

A novel dense granule molecule GRA24 has been identified. It can interact directly with p38*α* and lead to unusual phosphorylation of the host kinase [35]. In addition to the phosphorylation process, a recent proteomic study indicates that two proteins in the* Toxoplasma* motor complex are palmitoylated, glideosome associated protein (GAP45), and myosin A tail domain interacting protein (MTIP) and that palmitoylation of GAP45 and MTIP can regulate their cellular localization and function [36]. In addition, GAP45, one of the proteins that constitute the myosin XI motor complex required for motility of* T. gondii,* is phosphorylated at several sites. The transient phosphorylation of TgGAP45 at S163 and S167 appears to be important for glideosome assembly, and a TgGAP45 mutant in which these phosphorylation sites were mutated fails to interact with GAP50, the membrane anchor of the complex [37].

## 3. *Toxoplasma gondii *Modulates Host Cells Apoptosis

### 3.1. Apoptosis Pathways Targeted by* T. gondii*

Different studies in mouse and human cells have demonstrated that* T. gondii *modulates the apoptotic response of host cells, through downregulation [38] or upregulation of host-cell apoptosis [39]. The specific pathways, targeted by the parasite, may differ depending on the infection stage (acute or chronic), the virulence of the parasite strain, and the affected cell type. HSP 65 appears to contribute to immunity by preventing apoptosis of infected macrophages. A 1997 study by Hisaeda and collaborators showed that the high virulence* Toxoplasma* strain RH appears to have mechanisms that can enhance apoptosis in a culture of mouse peritoneal macrophages and in the mouse machrophage-derived J77A.1, namely, suppression of heat shock protein (HSP) 65 expression in infected cells [39]. A 1996 report by Khan et al. demonstrated that the avirulent* Toxoplasma *strain ME49 promotes apoptosis of mice CD4^+^ splenocytes via Fas-FasL interaction at 7 days after the infection [40]. Recently, Dincel et al. (2015) demonstrated also that this avirulent strain induces severe neurodegeneration in toxoplasmic encephalitis in mice. This severe inflammation is accompanied by a high expression of apoptosis mediators ADAMTS-13 (a metalloproteinase), caspase 3, caspase 8, caspase 9, TNFR1, and NO [41, 42]. Comparative analysis of these studies suggests that the virulent profile of the* T. gondii *strain is not a major determinant of the parasite's ability to interfere with apoptotic process. Indeed infection of murine astrocytes by the highly virulent RH strain led to decreased expression of the antiapoptotic proteins survivin, p53 upregulated modulator of apoptosis (PUMA), and Bcl-2 and increased expression of the proapoptotic protein Noxa in the early stage of infection [43]. In the brain of TE mice, neuron apoptosis was noted to be a result of primary injury due to parasite burden or resulted from the secondary response to microglia activation, which promotes neuron cell damage [44]. The latter hypothesis was confirmed by the study of Luo et al*.,* in which they found that infected microglia secreted the proinflammatory cytokines IL-1*β*, IL-6, TNF-*α*, and inducible NO synthase (iNOS). The inflammation generated is responsible for neuronal lesions in the brain of infected mice with reactivated TE [45].

Conversely,* T. gondii *has the ability to inhibit apoptosis in several murine and human host cells treated with a broad spectrum of proapoptotic stimuli [7, 38, 46, 47]. Presumably,* Toxoplasma gondii *interferes with different processes to inhibit apoptosis for maintaining chronic* Toxoplasma* infection.

#### 3.1.1. Blocking Activation of Proapoptotic Proteins

**Figure 2:**
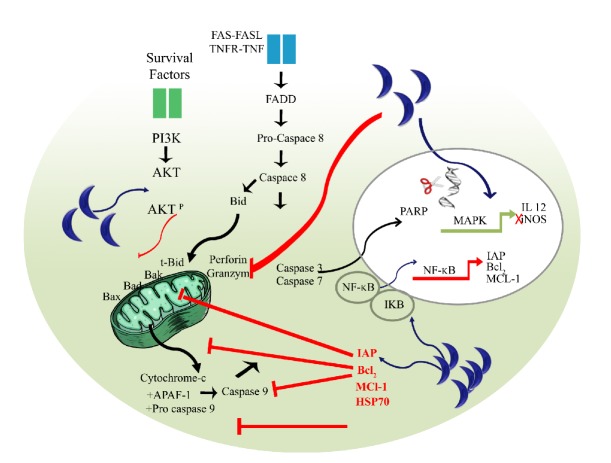
*Toxoplasma gondii *modulates mechanisms and signalling pathways of apoptosis.

*In vitro* studies have shown inhibition of mitochondrial cytochrome c release by virulent (type I) RH strain tachyzoites in MEFs (Mouse Embryonic Fibroblasts) [46, 48] and by avirulent (type II) NTE strain tachyzoites in human promyelocytic leukemia cells (HL-60) via a direct inhibition of caspases 3 and 9 [7]. But* Toxoplasma gondii *affects other apoptotic pathways. In the type I human B lymphoblastoid cell line (SKW6.4), the* Toxoplasma* RH strain induces an alteration of apoptosis triggered by Fas/CD95 path by degrading the procaspase 8 protein [49] (Figure 2).* T. gondii *has the ability to also downregulate TNFR1 or TNFR2 expression [50], as seen in RH-infected MRC5 human embryonic fibroblasts [50]. In addition to the major apoptotic pathways,* T. gondii *parasites have the capacity to affect other cell death pathways such as cytotoxic T lymphocytes (CTL) activities. RH-infected MLR murine T lymphoblasts and murine CD8^+^ T cells (M12-neo-1 cells) seem to be resistant to Perforin and Granzyme-mediated apoptosis (Figure 2). This inhibition of the apoptotic activity of CTLs by* T. gondii* infection [38, 49, 51] may involve a direct link between the inhibition of apoptosis and the association between the* T. gondii* vacuolar membrane and host cell mitochondria [52, 53]. Additionally,* Toxoplasma* interference with apoptosis may extend to targeting of the stage of DNA degradation.* Toxoplasma gondii* affects PARP expression in murine macrophage cells (RAW264.7 cells) infected by the avirulent NTE strain of* Toxoplasma* (Figure 2), decreasing PARP expression within 10 min and suppressing within 1 h of infection [54]. In addition, the atypical* Toxoplasma* TgCtwh3 can activate phosphorylation of STAT3 in infected human macrophages. This activation induces an overexpression of miR-17-92-miRNAs that inhibit the proapoptotic protein Bim, leading to the survival of the macrophage infected by this atypical strain [55]. The polymorphic form of ROP16 produced by this strain is similar to that produced by the type I* Toxoplasma *strain RH, so authors suggested that this ROP16-activated macrophage survival mechanism could be similar to that exerted by the canonical type I* Toxoplasma* strain [55].

The growth factors G-CSF and GM-CSF also seem to be targeted by* T. gondii* to manipulate apoptosis. Channon and collaborators demonstrated that both G-CSF and GM-CSF secreted by human fibroblastic cells infected with the avirulent PLK strain of* Toxoplasma* inhibited apoptosis of human neutrophils [56], and therefore* T. gondii*-induced G-CSF and GM-CSF secretion can promote neutrophil survival. Recently, Wang et al. demonstrated that the RH strain induces apoptosis in neural stem cells (NSCs) via the endoplasmic reticulum pathway. This finding was confirmed by a high expression of C/EBP homologue protein (CHOP), caspase 12, and activation of JNK pathway in NSCs cocultured with tachyzoites of RH* Toxoplasma* strain [57].

#### 3.1.2. Upregulating Antiapoptotic Proteins

Upregulation of antiapoptotic proteins includes expression of antiapoptotic Bcl-2 family [47, 58], downregulation of proapoptotic Bax proteins [48] (Figure 2), and the prevention of Bax/Bak activation by BH3-only family members [59]. In addition, the HSP70 family, an endogenous pleiotropic inhibitor of apoptotic cell death has been reported to also regulate apoptosis by influencing the apoptosis promoting activity of mitochondria [60, 61]. Heat shock is capable of inducing apoptosis and causes a cell initiate synthesis of heat-shock protein (HSPs). HSP70 derived from* T. gondii*-infected cells has been shown to be important for the control of host immune responses. Indeed, injection with* T. gondii *HSP70 caused an important reduction of parasite burden in various organs of B6 and BALB/c mice during both acute and chronic phases of toxoplasmosis [62]. Bcl-2 and HSP70 expressed by* Toxoplasma*-infected human monocyte infected cells (TH1) inhibited the mitochondrial permeability transition pore, blocked the release of both cytochrome c and apoptosis inducing factor protein (AIF) from the mitochondria, and inhibited ATP-mediated procaspase 3/9 [58] (Figure 2).

The antiapoptotic serine protease inhibitors Serpin B3 and B4 are significantly induced in acute monocytic leukemia-derived macrophages of the THP-1 lineage infected with the RH strain of* T. gondii* through activation of STAT6[63], and Serpin B3 and B4 may thus function as cellular factors involved in the parasite's regulation of the apoptosis pathway [64].

### 3.2. Apoptosis Signaling Pathway Targeted by* T. gondii*

#### 3.2.1. NF-*κ*B Pathway

The NF-*κ*B pathway consists of a family of primary transcription nuclear factors, conserved in all multicellular eukaryotic organisms and expressed in almost all cell types. These factors regulate the expression of antiapoptotic, proinflammatory cytokines. NF-*κ*B signaling is extremely important to the host immune response against* Toxoplasma* infection and regulates the direction of the host's immunity.

It has been described that* T. gondii* regulates apoptosis pathways through A1-a, an antiapoptosis member of the Bcl-2 family. This process may be an important proinflammatory event in acute host responses. It leads to an increase in parasite burden in the nonapoptotic cells [65]. The* Toxoplasma* RH strain upregulates also the expression of Mcl-1 (Figure 2) (another antiapoptotic Bcl-2 family member) and inhibitors of apoptosis (IAP) [7, 66]. Many of the cellular IAPs are known to be under NF-*κ*B regulation [67, 68], which promotes the host prosurvival machinery and proinflammatory expression proteins in mouse NIH 3T3 Balb/c fibroblasts [47]. The NF-*κ*B pathway plays a crucial role in modulating both innate and adaptive immune responses. NF-*κ*B activation is extensively considered to play an antiapoptotic function in cellular responses to diverse injurious stimuli [69]. In bone marrow-derived murine macrophages infected by the RH strain, p50, p65, and RelB were observed in response to parasite challenge during* in vitro *and* in vivo* studies [70, 71]. The importance of the NF-*κ*B pathway for regulating the immune response to survive acute* Toxoplasma* infection [72] was confirmed in a study using mice deficient in Bcl-3, a protein involved in the regulation of NF-*κ*B [73]. Using DNA arrays, expression of Bfl-1, IAP2, and TNFR1 was elevated over threefold in wild-type cells compared to mice deficient in the p65 subunit of NF-*κ*B [66]. The parasite-mediated induction of Bfl-1 might prevent host cell death primarily through the regulation of mitochondrial events such as membrane depolarization, cytochrome c release, and caspase-9 activation [74, 75] (Figure 2). IAP activation (1 and 2) enhances T cells activation [76]. A lack of NF-*κ*B activity could have an important effect in enhancing the expression level of survival genes [66]. In addition,* T. gondii-*infected mouse spleen cells display activation of antiapoptotic Bcl-2 family members but not proapoptotic pathways. The resistance of* T. gondii*-infected mouse spleen cells to apoptosis was attributed to the prevention of caspase-3 activity and PARP activation. This apoptosis inhibition was regulated by the activation of NF-*κ*B that promotes transcription of antiapoptotic genes (Figure 2). In this case, the expression of NF-*κ*B is correlated with antiapoptotic protein expression after* T. gondii *infection [77]. A recent study has shown that the NF-*κ*B pathway can be implicated in the activation of the apoptotic process in human leukaemia cell line. The* T. gondii* ME-49 strain can inhibit NF-*κ*B activation via upregulation of the protein levels of the A20 ubiquitin chain enzyme that inhibits NF-*κ*B by inhibiting the TCR signaling pathway in infected Jurkat T-cells and Molt-4 T-cells [78].

#### 3.2.2. MAPKinase (Mitogen-Activated Protein Kinase) Pathways

In addition to the NF-*κ*B pathway, MAPKinase pathways are also involved in activation of both innate and acquired immune responses during infection by* T. gondii *[77].* T. gondii *induces P38 MAPKinase autophosphorylation in mouse macrophages, promoting IL-12 production [79, 80] (Figure 2). The* Toxoplasma* mitogen-activated protein (*Tg*-MAPK) has been identified as a virulent factor regulating host immunity response and* Toxoplasma* tachyzoite proliferation [81]. The* Tg*-MAPK controls IFN-*γ*-mediated iNOS expression and NO production and promotes activation of host cell p38 MAPK activation, thereby limiting host iNOS production [81] (Figure 2). The inhibition of apoptosis observed in BeWo cells (human placental cells) after infection with RH strain is associated with the increased phosphorylation of the antiapoptotic ERK1/2 protein [82]. Recently, the study by Cao and collaborators demonstrated that in African green monkey kidney cells (Vero cells) infected by type I* Toxoplasma* strain, the* Toxoplasma* MAPK1, the orthologue of mammalian MAPK P38 *α*, is involved in bradyzoite differentiation and in asexual development of* T. gondii *[83].* Toxoplasma* can also interact with p38*α* via GRA24, promoting host kinase activation, leading in most cases to the host death [84].

#### 3.2.3. JNKinase (c-Jun N-Terminal Kinases) Pathways

The JNKinase pathway is also involved in activation and/or inhibition of apoptosis mechanisms during infection by* T. gondii*. The role of JNK, as either a proapoptotic or prosurvival mediator, is dependent upon the magnitude and duration of JNK activation and appears to be cell-type specific [85], while the transcription factor NF-*κ*B has recently been shown to negatively regulate JNK activation [86, 87]. Carmen et al. [88] showed that the JNK pathway does not involve the NF-*κ*B signaling pathway* Toxoplasma*-infected human Hela cells. In infected mouse bone marrow-derived macrophages and human peripheral blood monocytes,* Toxoplasma* induced the B7-2 protein, which initiates T cell proliferation. B7-2 production is mediated via the JNK signaling pathway. The effective role of JNK signaling pathway is not yet clear during* Toxoplasma* infection [89].

The JNK pathway is implicated also in the upregulation of c-Myc (host cell growth) in* Toxoplasma*-infected human foreskin fibroblasts (HFFs). It is reported that* T. gondii* may be manipulating c-Myc to prevent both host cell and parasite apoptosis, enabling* Toxoplasma* to survive and proliferate inside the host. Similarly, c-Myc-mediated regulation of host immune function could serve as protection from host immune recognition [90]. The ASK1/JNK is also involved in activation of apoptosis trophoblasts in the mouse model during acute* T. gondii *infection [91].

#### 3.2.4. PI3K/PKB/Akt Pathways

Phosphoinositide 3 kinase (PI3K) is a family of signal transducing enzymes implicated in various cellular functions, such as growth, proliferation, and cell survival, ensuring the maintenance of homeostasis. This pathway is activated during* T. gondii *infection and is essential for the invasion and proliferation of the parasite in the host cell. The phosphoinositide 3-kinase pathway and the immediate downstream effector protein kinase B (PKB/Akt) play important roles in cell survival and apoptosis inhibition.* Toxoplasma gondii *activates these pathways in a dose-dependent manner via toll-like receptors TLR2 and TLR4 [92]. PI3k / AKT decreases the expression of the nicotinamide adenine dinucleotide phosphate (NADPH) oxidase 4 (NOX4) gene, which then reduces the amount of Reactive Oxygen Species (ROS) in the infected cell and consequently provides a suitable environment for the proliferation of* T. gondii *in host cell [93]. ROS plays an essential role in the immune response against pathogens, such as bacteria and intracellular parasites. One of the major sources of ROS is NADPH and more specifically NOX 4. Moreover, it was described that Nox4-mediated ROS generation plays a central role in macrophage migration inhibitory factor (MIF) production and resistance to* T. gondii* infection in the mouse model [94]. Likewise, this pathway is involved in the regulation of the secretion of Il-23 and Il-12 in* Toxoplasma*-infected Jurkat cells [95]. This suggests that both the Il-23 and Il-12 are implicated in the resistance of* Toxoplasma* infection [34].

It was reported that* Toxoplasma* infection of mouse macrophages activates PKB/Akt* in vivo* and* in vitro*. This pathway promotes inhibition of the apoptosis challenge via host G_i_-protein-dependent PI 3-kinase signalling [96]. In addition,* T. gondii *inhibits apoptosis mechanism also via this signaling pathway in THP-1 cells and splenocytes. The phosphorylation of AKT protein promotes phosphorylation of Bad protein which can prevent apoptosis activation by blocking Bax translocation, cytochrome c release, and caspase 3/9 activation [97] (Figure 2).

## 4. Conclusion

*Toxoplasma gondii* manipulates many signalling pathways intended for host defense to achieve persistence in host cells. Among the host signalling pathways targeted by* Toxoplasma* are apoptotic pathways. This response induces, in most cases, a local inflammatory reaction as in the case of TE. Most studies are described in the mouse model. It would be important to determine the difference of* T. gondii* strains effect on the inhibition of proapoptotic pathways and activation of antiapoptotic pathways in* in vitro* coculture system of different cells that can be infected by* T. gondii* [98]. This model allows us to study the interaction of different host cells in the presence of the parasite. Thus, the caspase-dependent apoptotic pathway has been well studied. Further research on the effect of* T. gondii* virulence proteins could be focused on pathways not depending on caspase. New molecular biology and proteomic technics will permit the identification of the activation, inhibition, and interaction of, and between, several proteins responsible for the signalling pathways involved in the persistence of* T. gondii* in host cells.
